# Streptavidin-coated gold nanoparticles: critical role of oligonucleotides on stability and fractal aggregation

**DOI:** 10.3762/bjnano.8.1

**Published:** 2017-01-02

**Authors:** Roberta D'Agata, Pasquale Palladino, Giuseppe Spoto

**Affiliations:** 1 Consorio Interuniversitario “Istituto Nazionale Biostrutture e Biosistemi”, c/o Dipartimento di Scienze Chimiche, Università di Catania, Viale Andrea Doria 6, I-95125, Catania, Italy; 2 Dipartimento di Scienze Chimiche, Università di Catania, Viale Andrea Doria 6, I-95125, Catania, Italy

**Keywords:** biosensors, DNA, gold nanoparticles, nanoparticles aggregation, plasmonics, streptavidin

## Abstract

Gold nanoparticles (AuNPs) exhibit unique properties that can be modulated through a tailored surface functionalization, enabling their targeted use in biochemical sensing and medical diagnostics. In particular, streptavidin-modified AuNPs are increasingly used for biosensing purposes. We report here a study of AuNPs surface-functionalized with streptavidin-biotinylated oligonucleotide, focussing on the role played by the oligonucleotide probes in the stabilization/destabilization of the functionalized nanoparticle dispersion. The behaviour of the modified AuNP dispersion as a consequence of the competitive displacement of the biotinylated oligonucleotide has been investigated and the critical role of displaced oligonucletides in triggering the quasi one-dimensional aggregation of nanoparticles is demonstrated for the first time. The thorough understanding of the fundamental properties of bioconjugated AuNPs is of great importance for the design of highly sensitive and reliable functionalized AuNP-based assays.

## Introduction

Gold colloids have been the focus of research for many decades because of their intriguing electronic and optical properties, depending on the size and shape of gold nanoparticles (AuNPs) [1], which support several biomedical and pharmaceutical applications [2]. The functionalization of AuNPs with biologically relevant ligands has led to dramatic progresses in both living cells as well as biomolecular diagnostic assays [3–5]. In particular, optical sensing exploiting the surface plasmon resonance (SPR) effect has been widely investigated and plays a significant role in biomolecular detection [6–7]. In this context, it has been shown that AuNPs can be also employed to enhance responses from SPR experiments aimed at detecting biomolecular interactions occurring at a flat metal–solution interface. Such SPR experiments are designed to reveal changes in conditions required to couple electromagnetic radiation to surface plasmons (SPs) propagating along the interface between the flat metal surface and dielectric. The signal enhancement produced when AuNPs are used in assays is a consequence of the large variation of the local dielectric constant caused by AuNPs [8]. In fact, the interaction between propagating and localized SPs established when nanoparticles are few nanometers far from the flat metal surface reinforces the local electric field [9–10]. The ultrasensitive detection of nucleic acids has been recently achieved by using streptavidin (SA)-conjugated AuNPs and SPR imaging (SPRI) [11–12]. In this case, the enhanced sensitivity enables the detection of point mutations in non-amplified human genomic DNA with attomolar sensitivity [13], thus offering an excellent cost-effective alternative to time consuming and prone to sample contamination nucleic acid amplification protocols [14]. In this context, the interaction of SA-conjugated AuNPs with large DNA fragments immobilized on the surface of SPRI sensors has been hypothesized to induce an AuNP aggregation process which could contribute to further enhance the sensitivity of nanoparticle-enhanced SPRI DNA detection assays [12]. Such hypothesis has motivated further studies on oligodeoxyribonucleotide (ODN)-functionalized SA-conjugated AuNPs. SA-conjugated AuNPs have not been so widely studied as thiol-conjugated AuNPs. Nevertheless, ODN-functionalized SA-conjugated AuNPs are largely used for different purposes including biosensing [15–17].

Results from spectroscopic, dynamic light scattering (DLS), zeta-potential (ζ), transmission electron microscopy (TEM) and SPR investigations of ODN-functionalized SA-mediated AuNPs are here presented with the aim to provide fundamental information useful to improve performances of biosensing assays using SA-mediated AuNPs. The data shown here demonstrate that the electrostatic repulsion provided by the negatively charged ODN moieties contributes to the final AuNP stabilization against aggregation, as already reported for thiol-mediated conjugation of citrate-stabilized AuNPs [18–19]. In addition, it is demonstrated that ODNs displaced from the functionalized AuNP surface lead to fractal aggregation of nanoparticles, which is here described for the first time on the basis of ODN displacement from the AuNP surface, in agreement with the previously reported depletion attractions between hard colloidal silica spheres immersed in a non-adsorbing solution of double-stranded DNA [20], according to the Asakura–Oosawa model for depletion flocculation in colloidal dispersions [21–25]. This evidence support the hypothesis that surface deposited single or double stranded DNA, if released in solution, could cause local aggregation of AuNPs that could be exploited to increase the sensitivity of AuNP-enhanced nucleic acid detection assays.

## Results and Discussion

### Functionalization of nanoparticles

The adsorption of proteins on nanoparticles has been widely investigated over the last decade [26]. In particular, it has been demonstrated that the interaction between proteins and citrate-stabilized AuNPs occurs through a mechanism involving carboxylate–ammonium interactions established between citrate and lysine or histidine amino groups on the protein surface. The mechanism also includes contributions from steric or hydrophobic interactions with the nanoparticle surface adlayer [27].

**Figure 1:**
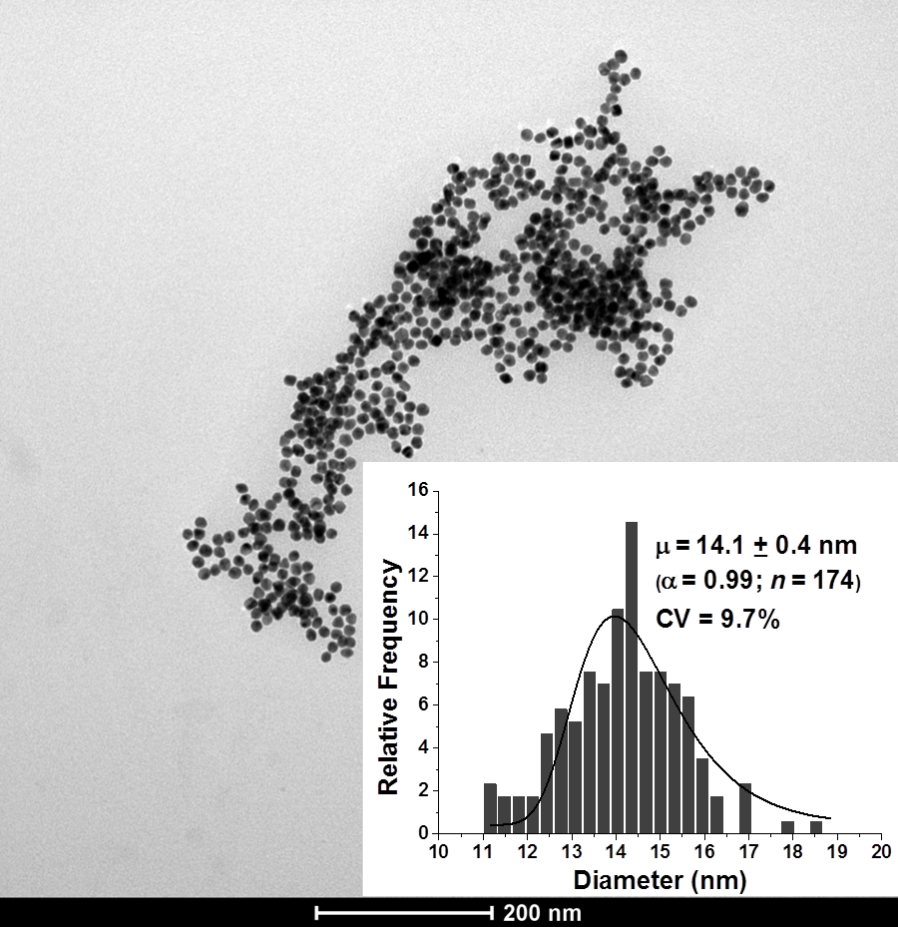
TEM micrograph and dimensional dispersion histogram (inset) of bare AuNPs.

Monodispersed spherical AuNPs with an average diameter of 14.1 ± 0.4 nm (%CV = 9.7) with a surface area availability per nanoparticle of about 625 nm^2^ were used for our experiments (Figure 1).

Considering that SA has a hydrodynamic diameter of 5 nm [28–29] and a projected surface area of approximately 25 nm^2^, a maximum of about 25 SA molecules per nanoparticle can contribute to the formation of an adsorbed monolayer.

**Figure 2:**
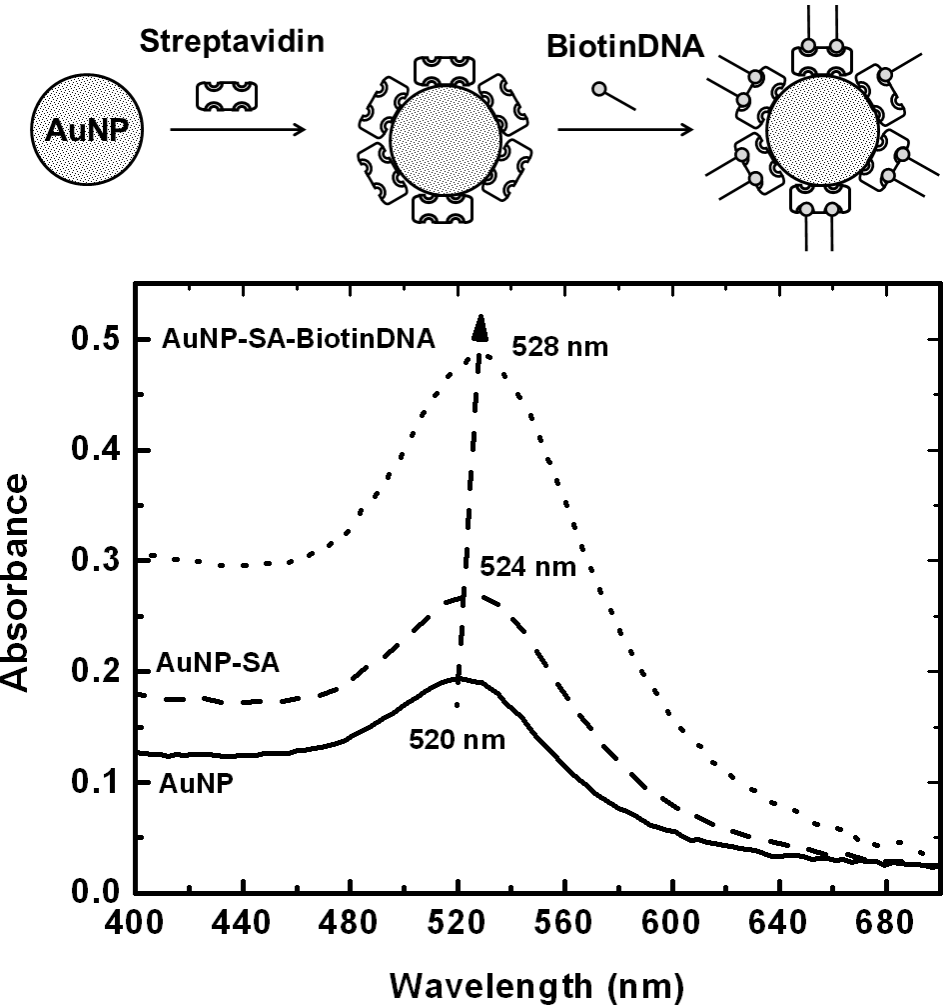
Absorbance spectra of bare AuNPs and SA coated nanoparticles before (AuNP-SA) and after (AuNP-SA-BiotinDNA) the adsorption of biotinylated oligonucleotides. The change of the intensity of the peaks reflects the different concentration of colloidal dispersions after each functionalization step.

AuNP-SA and AuNP-SA-BiotinDNA have been investigated by monitoring the optical properties of colloidal suspensions [1]. Figure 2 shows the absorbance spectra of AuNP dispersions before and after the nanoparticle functionalization. Bare AuNPs exhibit a localized SPR peak at 520 nm that shifted to 524 and 528 nm upon adsorption of SA on the nanoparticle surface and subsequent conjugation with BiotinDNA, respectively.

The shift is a consequence of changes in the local dielectric constant and effective thickness of the layer adsorbed on the AuNP surface [30]. No significant broadening of peaks is observed after the functionalization steps, indicating that particles did not appreciably aggregate.

The 4 nm shift displayed by absorption spectra after the absorption of SA can be used to predict the mass of protein absorbed per unit area [31]. The prediction is based on the assumption that AuNP-SA can be depicted as a sphere with a homogeneous spherical shell. On this basis, the spectral shift (Δλ) is given by Equation 1:

[1]
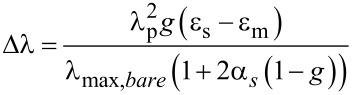


where ε_s_ and ε_m_ represent the dielectric constant of the shell (SA) and the surrounding medium (water), respectively, λ_p_ is the bulk metal plasmon wavelength (131 nm for gold [32]), λ_max,_*_bare_* is the wavelength of maximum absorption for AuNPs (520 nm, Figure 2), *g* is the fraction of nanoparticle that is shell, and α_s_ = (ε_s_ − ε_m_)/(ε_s_ + 2ε_m_). *g* can be obtained from Equation 1 with Δλ = 4 nm by assuming a refractive index (*n*) of 1.334 for the medium (water) [33] and a refractive index of 1.47 for SA [34] (ε*_m_* = *n*_water_^2^ and ε*_s_* = *n*_SA_^2^). The coating thickness (*s*), that is obtained from Equation 2:

[2]
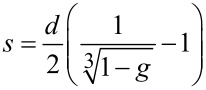


where *d* is AuNP diameter (Table 1), that can be used to calculate the coverage (Γ, mass of SA per unit area) of AuNP-SA using Equation 3 [35]:

[3]
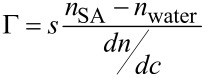


By using the value 0.212 cm^3^ g^−1^ for the refractive-index increment (*dn*/*dc*) of SA [34] a coverage of 4.5 mg m^−2^ of SA, corresponding to about 30 SA molecules per nanoparticle, is obtained.

SA is a tetrameric protein with four identical subunits arrayed in D2 symmetry, and each subunit has a biotin binding pocket, therefore it is to be expected that no more than two of the four biotin binding pockets are accessible by BiotinDNA per surface-adsorbed SA molecule. On this basis, it can be estimated that up to ≈60 BiotinDNA molecules were immobilized per AuNP.

Properties of dispersed nanoparticles were also determined by DLS and ζ-potential (Table 1) [36].

**Table 1:** Data from TEM, DLS and zeta potential characterization of nanoparticles (mean ± SD; *n* = 6 samples × 3 replicate). 0.1 nM nanoparticle dispersion in H_2_O.

Sample	Physical diameter (nm) %CV^a^	ζ (mV)^b^	z-ave (nm)^c^	PDI^d^

AuNP	14.1 ± 0.4 9.7%	−38.9 ± 1.7	19.4 ± 1.8	0.22 ± 0.07
AuNP-SA	–	−60.3 ± 3.9	31.1 ± 0.7	0.41 ± 0.01
AuNP-SA-BiotinDNA	–	−48.7 ± 2.31	39.8 ± 0.9	0.48 ± 0.02

^a^TEM-measured diameter of the gold core. %CV: Coefficient of Variation. ^b^Zeta potential. ^c^Intensity-weighted harmonic mean hydrodynamic diameter (z-average diameter). ^d^Polydispersity index from DLS measurements.

**Figure 3:**
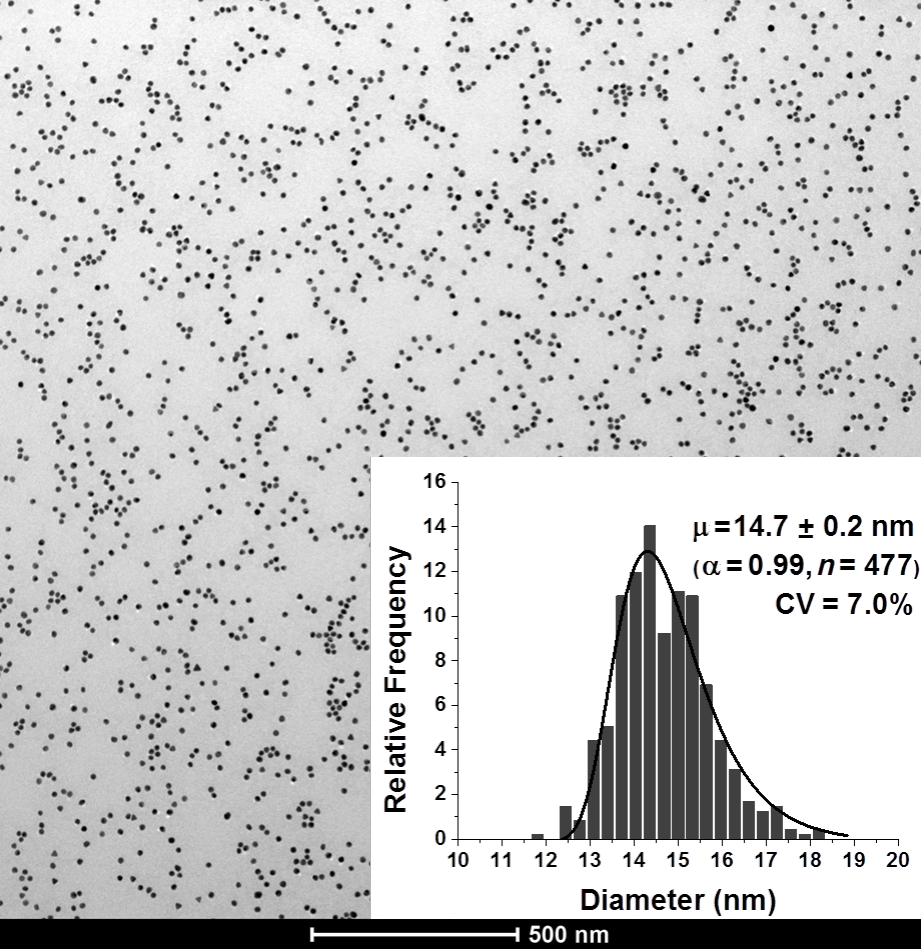
TEM micrograph and dimensional dispersion histogram (inset) of AuNP-SA-BiotinDNA.

Bare AuNPs with an intensity-weighted harmonic mean hydrodynamic diameter (z-average) [37] of 19.4 ± 1.8 nm showed a z-average value of 31.1 ± 0.7 nm after functionalization with SA. The increase in diameter corresponds to a 5.8 nm thick adlayer compatible with the adsorption of an SA monolayer [28]. AuNP-SA-BiotinDNA shows a z-average of 39.8 ± 0.9 nm which corresponds to a further increase of 4.3 nm of the adlayer thickness, which is consistent with the contribution of the 11-mer BiotinDNA to the hydrodynamic size of the functionalized nanoparticles [38–39]. The AuNPs hydrodynamic diameter is larger compared to TEM-measured diameter. This evidence, combined with the PDI value of 0.22 ± 0.07 indicating a relatively narrow distribution of AuNPs, allow us to conclude that the number of agglomerates in the sample is small. In fact, while the scattering intensity is strongly affected by the radius (*R*) of the scattering particle (*I* 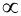 *R*^6^) a small number of agglomerates have a limited impact on TEM [40]. The PDI increased after AuNPs functionalization thus indicating an increase of polydispersity. Interestingly, the introduction of SA-BiotinDNA produced more favourable conditions for TEM analysis of dispersed nanoparticles, as shown in Figure 3.

The more negative ζ-potential of both AuNP-SA and AuNP-SA-BiotinDNA compared to citrate-stabilized AuNPs ζ-potential (Table 1) contributes to improving the stability of functionalized nanoparticles dispersions. Nanoparticles modified with biotinylated oligonucleotides are spontaneously redispersed because of steric and electrostatic repulsion provided by the introduction of negatively charged oligonucleotide moieties.

### Competitive displacement of biotinylated oligonucleotide from AuNP-SA-BiotinDNA

**Figure 4:**
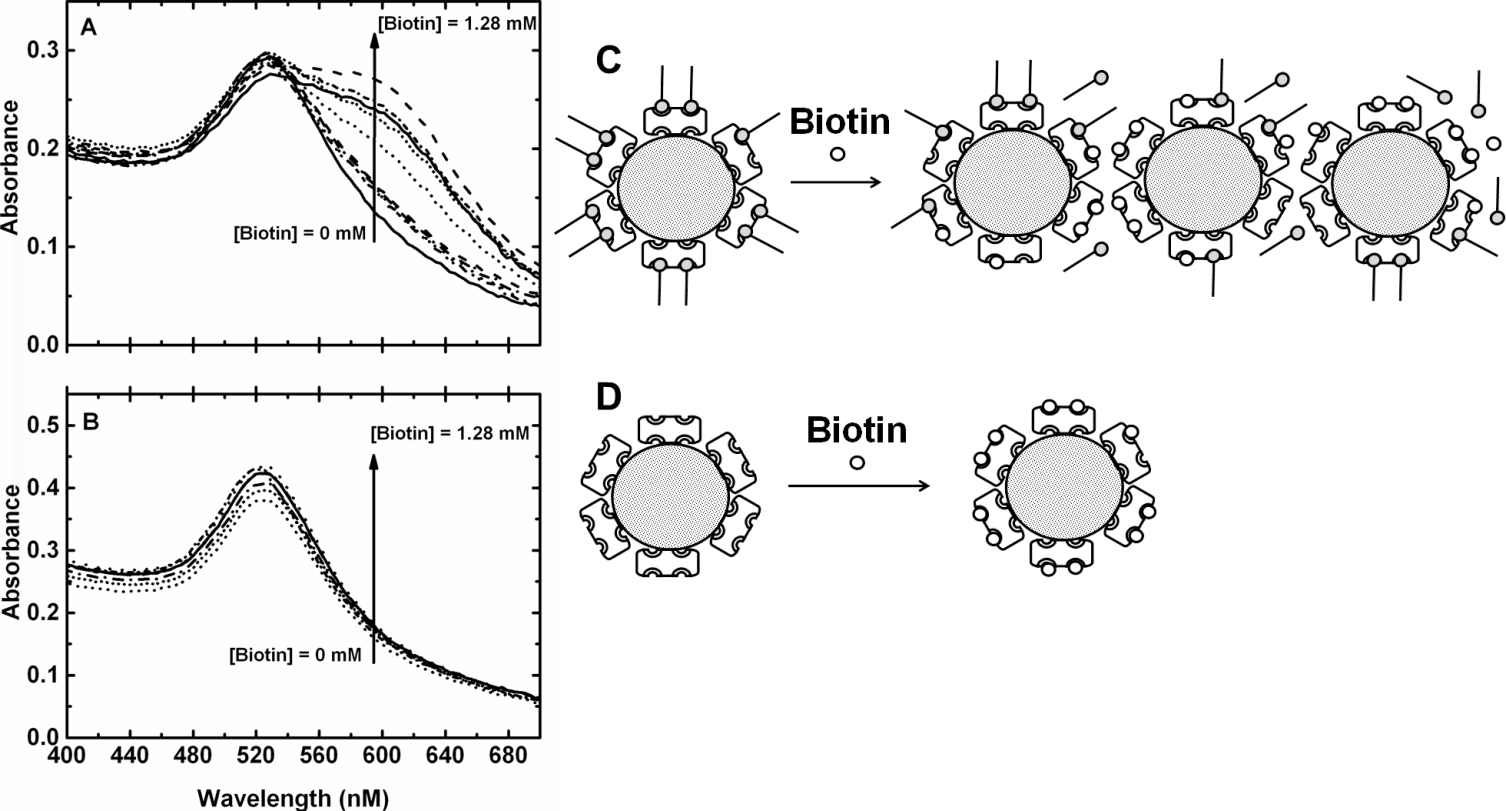
Absorbance spectra (400–700 nm) of AuNP-SA-BiotinDNA (A) and AuNP-SA (B) titred with free-biotin solution. Concentrations of free-biotin, from bottom to top, are 0, 0.01, 0.02, 0.04, 0.08, 0.32, 0.64, 1.28 mM. The effect of free-biotin titration of AuNP-SA-BiotinDNA (C) and AuNP-SA (D).

In order to investigate the role played by the biotinylated oligonucleotide in the stabilization of functionalized AuNPs and to acquire deeper knowledge of general properties of AuNP-SA-BiotinDNA, the competitive displacement of the biotinylated oligonucleotide from functionalized AuNPs was carried out. The dissociation of the very strong streptavidin–biotin complex (*K*_d_ ≈ 4 × 10^−14^ M) has been widely investigated both in homogeneous solution as well as at the solid–liquid interface [41–47]. When confined at the solid–liquid interface the whole interaction is influenced by factors that significantly affect the kinetics of the reaction. In particular, rate constants of the streptavidin–biotin dissociation in solution are smaller by a factor ranging from 10 to 10^2^ than on the surface [42]. The kinetics of the interaction between biotinylated-oligonucleotide and avidin on the surface of SU-8 microparticles shows a dissociation constant of 7 ± 3 × 10^−12^ M [48] that is higher than that measured in solution for the same equilibrium. However, in spite of that, the study of the dissociative equilibrium of streptavidin–biotin interacting on the surface requires the competitive displacement of the linked biotin molecules by free-biotin at the mM concentration to be performed [42,45,47]. Therefore, the competitive displacement of biotin-labeled oligonucleotide from AuNP-SA-BiotinDNA by free biotin was performed in our case. The addition of an increased amount of free biotin to AuNP-SA-BiotinDNA dispersions caused a gradual decrease of the intensity of the characteristic plasmon band at 528 nm while a plasmon band around 600 nm gradually emerged (Figure 4A). The process resulted in a characteristic colour change of the dispersion (from red to blue-violet) which is associated with nanoparticles aggregation (Figure 4C) [49–50]. When the same amount of free biotin was added to AuNP-SA dispersions (i.e., nanoparticles with no immobilized oligonucleotides) no similar changes were observed in the absorbance spectrum (Figure 4B) where one single absorption peak at 524 nm is displayed, thus demonstrating that AuNP-SA are stable in the presence of a large amount of free biotin (Figure 4D). These results suggest that the aggregation of AuNP-SA-BiotinDNA in contact with free biotin is caused by the displaced BiotinDNA and is not dependent on the high concentration of free biotin (up to 1.28 mM).

Displaced BiotinDNA brings negatively charged oligonucleotide strands in solution thus altering the electrostatic environment around colloidal nanoparticles and providing a new source for hydrophobic interactions with AuNP-SA [51]. In addition, the removal of BiotinDNA from the surface of AuNP-SA-BiotinDNA modifies the contribution of steric effects on the stabilization of the dispersion. This complex imbalance of stabilizing effects does not occur in AuNP-SA dispersions with free biotin (Figure 4D).

**Figure 5:**
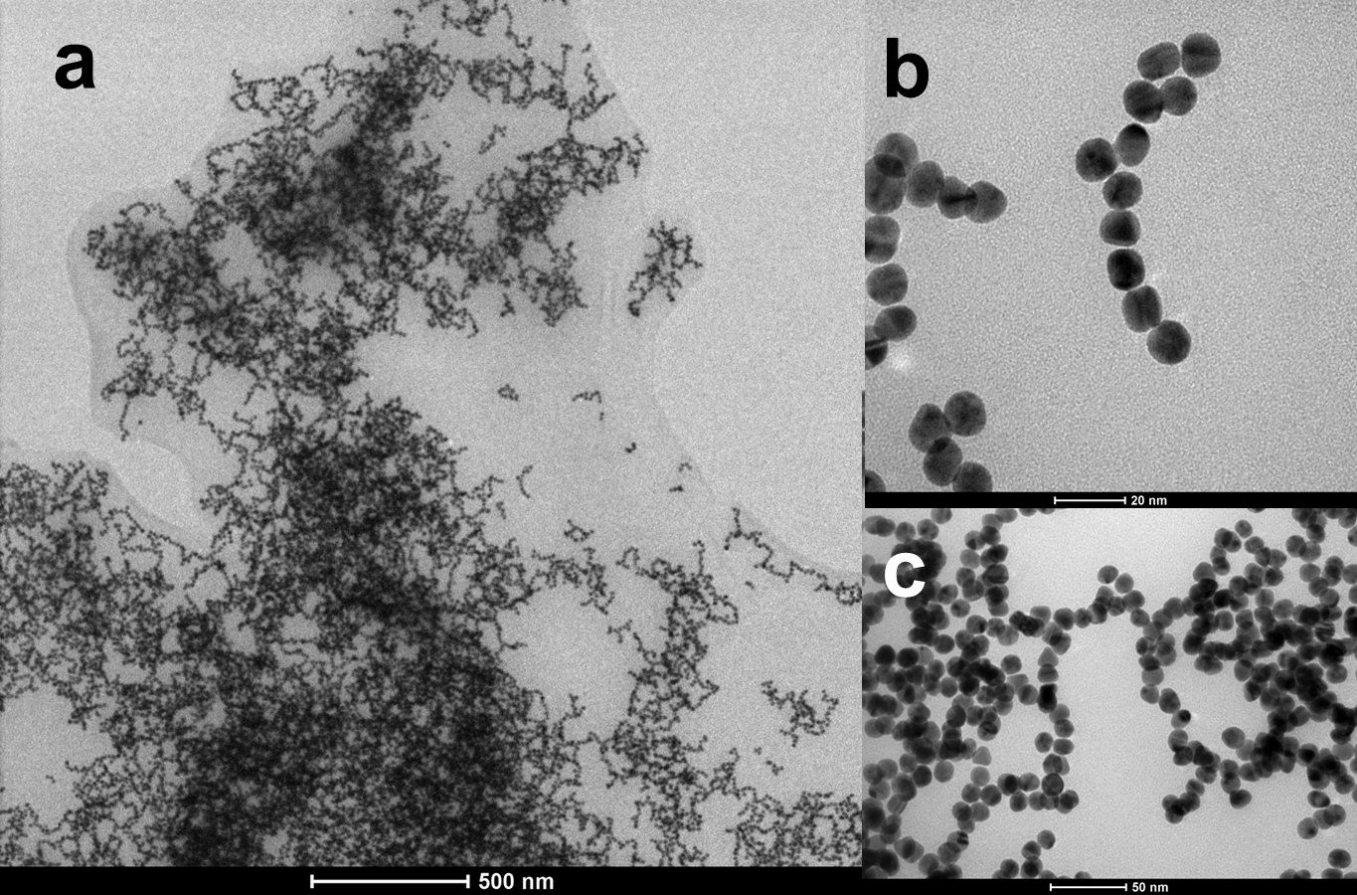
TEM micrographs of AuNP-SA-BiotinDNA after BiotinDNA displacement by free biotin (1.28 mM). TEM micrographs (a), (b), and (c) show aggregated nanoparticles with different magnification factors.

The massive aggregation of AuNP-SA-BiotinDNA after BiotinDNA displacement by free biotin was also revealed by TEM. Figure 5 shows large branched linear aggregates with variable dimensions. It is well known that drying a drop casted nanoparticle suspension on TEM grids often introduces artifacts [52]. In our case, the formation of aggregates in the liquid dispersion is demonstrated by the plasmon band at 600 nm observed in absorbance spectra of AuNP-SA-BiotinDNA after displacement by free biotin (Figure 4A). A red-shifted contribution to the gold nanoparticle plasmon band has been consistently observed from dispersions of AuNPs linear aggregates [53–55]. In addition, it has been demonstrated that bands generated by linear aggregates allow to discriminate between linear and spherical aggregates [53].

**Figure 6:**
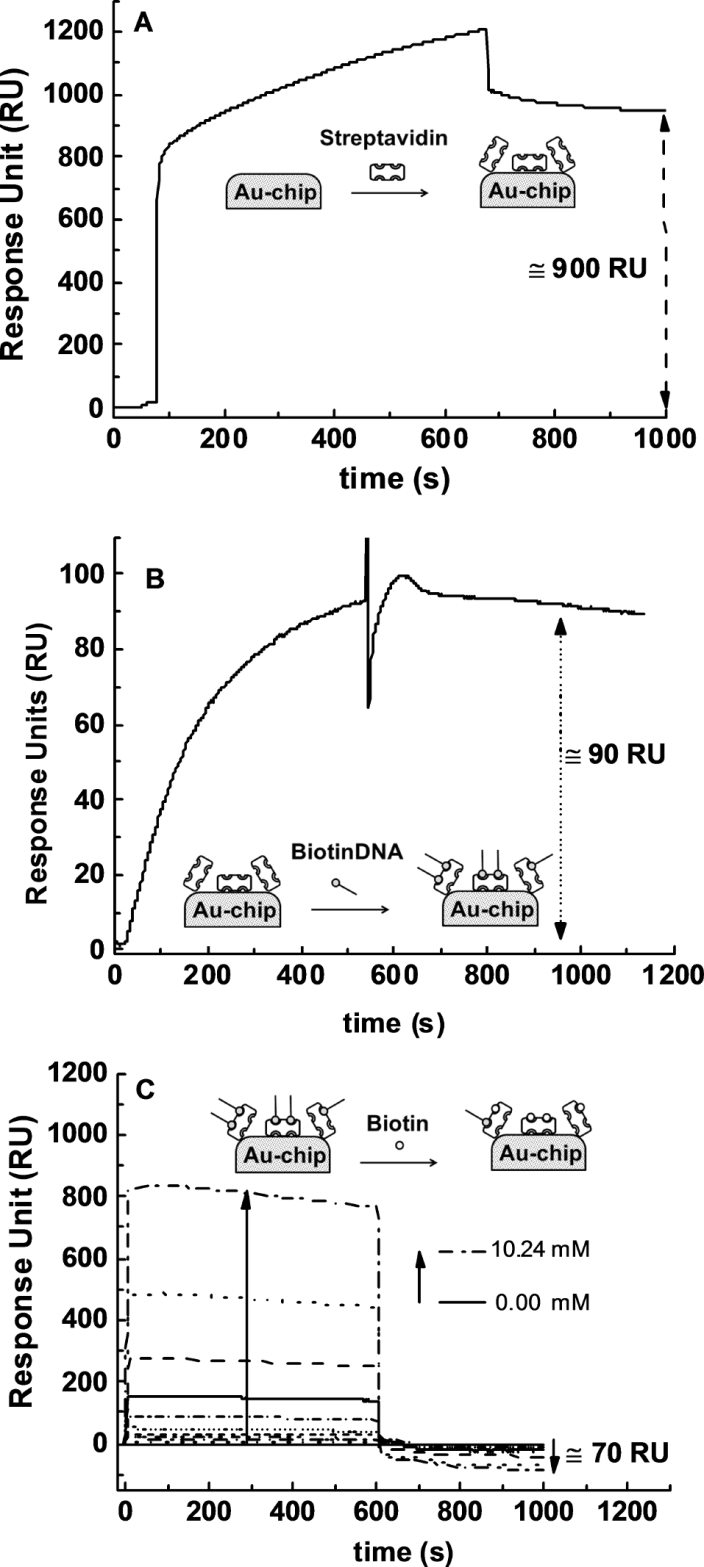
SPR data showing the competitive displacement of BiotinDNA by free biotin. (A) Streptavidin covalent immobilization on gold chip. (B) Streptavidin-BiotinDNA complex formation. (C) Competitive displacement of BiotinDNA by the interaction of increasing amount of free-biotin with surface-immobilized SA-BiotinDNA.

We confirmed the displacement of BiotinDNA from the SA-BiotinDNA complex by using SPR (Figure 6). Increasingly concentrated solutions of free biotin were adsorbed over SA-BiotinDNA immobilized on the SPR chip gold surface. The immobilization of BiotinDNA on surface-immobilized SA produced an SPR signal of about 90 RU (Figure 6B) while a negative shift of about 70 RU was observed after the competitive interaction with free-biotin (Figure 6C) that succeeded in displacing the immobilized BiotinDNA.

To better understand if the observed aggregation of AuNP-SA-BiotinDNA is a consequence of SA-BiotinDNA desorption from the surface of the functionalized nanoparticles, the interaction of BiotinDNA with bare AuNPs was also investigated. In fact, the direct interaction of the displaced BiotinDNA with the AuNP surface is to be taken into account if the whole SA-BiotinDNA complex instead of only BiotinDNA desorbs from AuNP-SA-BiotinDNA. The interaction of single-stranded DNA with citrate-stabilized AuNPs has been widely investigated and controversial mechanisms have been proposed for this process [51,56]. The discovery of a different propensity of single- or double-stranded oligonucleotides to adsorb onto citrate-stabilized AuNPs [57] led to further investigate the behaviour of ssDNA, which is adsorbed on AuNP surface more easily than dsDNA. Both citrate-stabilized AuNPs and oligonucleotides are negatively charged, therefore ssDNA adsorption is linked to the long-range electrostatic repulsion, which is related to the Debye length and is strongly influenced by salt concentration. The adsorption mechanism has been originally based on the Derjaguin–Landau–Verwey–Overbeck (DLVO) theory [58] that does not take into account both the base-sequence-dependent [59] and the salt-type-dependent adsorption. In particular, the latter has stimulated much research into the contribution of the hydrophobic effect which has been claimed to play a dominant role in ssDNA adsorption [51].

**Figure 7:**
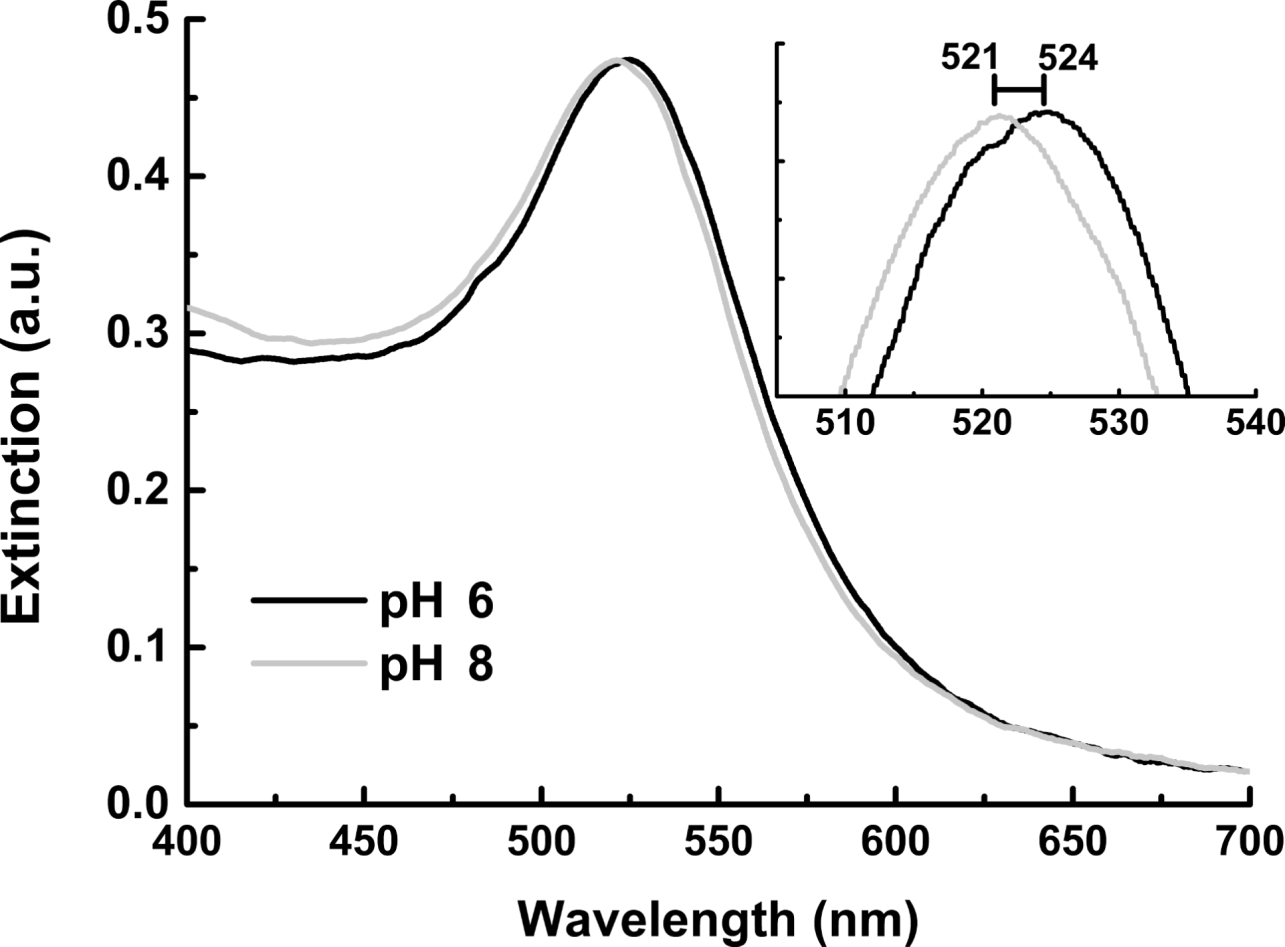
Normalized extinction spectra (400–700 nm) of AuNPs after the non-specific adsorption of BiotinDNA (no streptavidin) at different pH values.

The incubation of BiotinDNA with bare AuNPs at pH 6 produced a plasmon band at 524 nm (Figure 7) thus testifying the adsorption of the oligonucleotide sequence onto the nanoparticle surface. The large red-shift of the plasmon band (Figure 4A) attributed to the nanoparticle linear aggregation caused by BiotinDNA displacement is not observed in this case.

At pH 6 an almost complete deprotonation of citrate carboxyl groups is observed (citrate p*K*_a_ values are 3.2, 4.8, 6.4). It has been demonstrated that the electrostatic barrier created by surface citrate can be tuned by modifying the pH value thus modulating negative charges onto the nanoparticle surface [56]. In our case the pH dependence was confirmed by replicating non-specific adsorption experiments at pH 8. As expected, an almost negligible shift of the plasmon band (521 nm), which is a consequence of the disfavoured adsorption of BiotinDNA onto the more negatively charged citrate-stabilized AuNPs at higher pH, was observed in this case.

**Figure 8:**
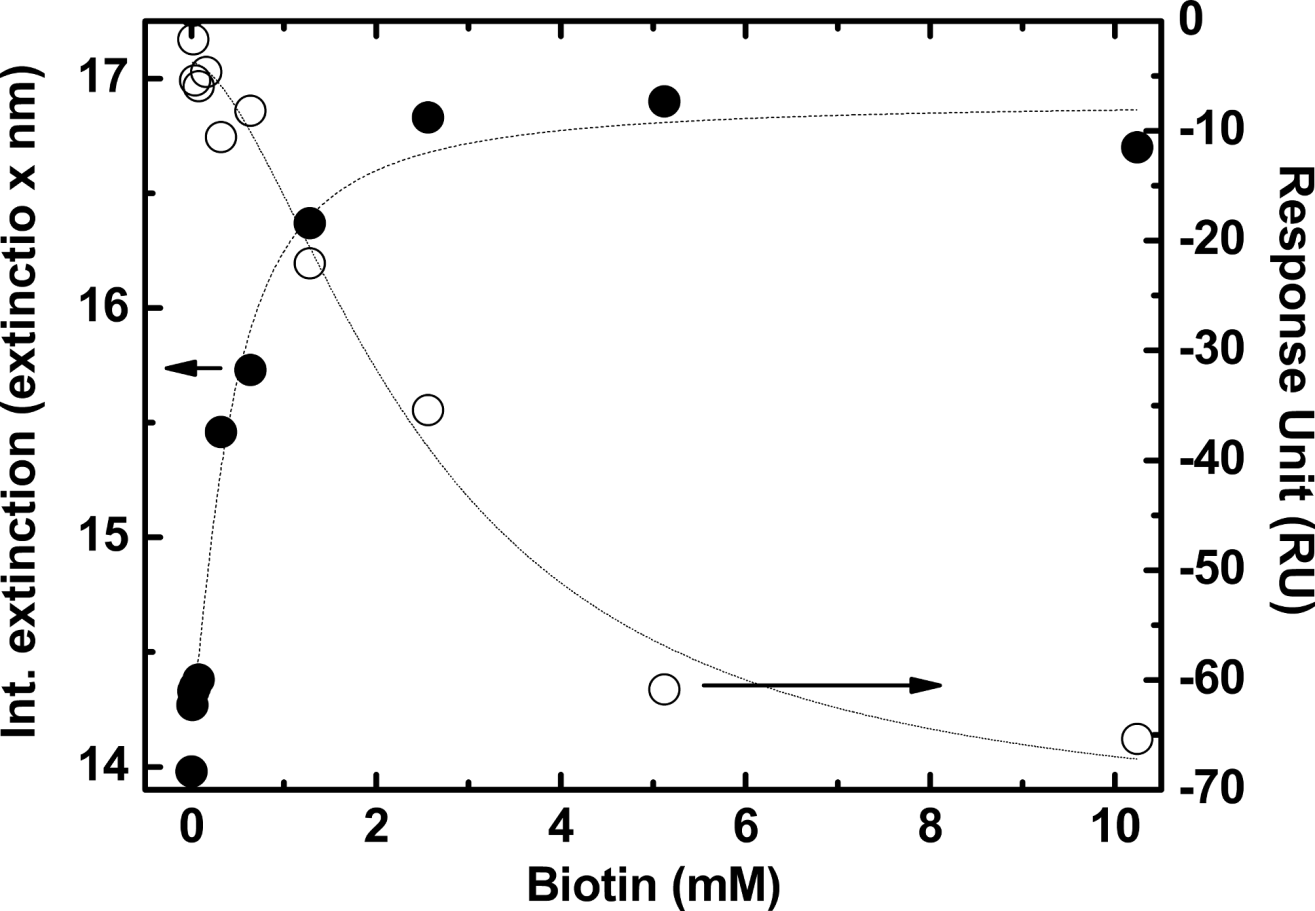
BiotinDNA displacement from AuNP-SA-BiotinDNA (●) and SA-BiotinDNA immobilized on SPR gold sensor chip (○) by free-biotin represented by the integrated extinction obtained from spectra shown in Figure 4A (left axis) and by SPR data shown in Figure 6C (right axis), respectively.

Different methods for detecting aggregation or agglomeration of gold nanoparticles have been investigated. Most of them rely on the integrated extinction, which increases with the degree of aggregate formation in agreement with theoretical principles based on the different contribution of transversal and longitudinal surface plasmons [60–61]. The integrated extinction between 400 and 700 nm for AuNP-SA-BiotinDNA dispersions incubated with differently concentrated free biotin are shown in Figure 8 (closed circles).

The graph shows that increased BiotinDNA displacement causes flocculation with a saturation of the integrated extinction, which is obtained after incubation with free biotin at concentrations higher than 2.56 mM. The competitive displacement of BiotinDNA by 10 µM free biotin is enough to cause detectable changes in the extinction spectrum thus testifying the important contribution of the displaced oligonucleotide in triggering processes leading to gradual flocculation. These results also suggest that the destabilization of AuNP-SA-BiotinDNA starts at the early stage of BiotinDNA displacement and does not require the SA binding sites saturation with free-biotin. Since experiments carried out (Figure 4B and Figure 7) demonstrated that neither the highly concentrated free biotin solution nor the direct adsorption of BiotinDNA on AuNPs can be responsible for the gradual aggregation of AuNP-SA-BiotinDNA, two possible mechanisms could be responsible for the observed aggregation process. One is cross-bridging that would occur if BiotinDNA simultaneously adsorbs to surfaces of separate colloidal particles. However, displaced BiotinDNA cannot interact with SA with its biotin units since SA pockets are occupied by free biotin. The other more reasonable mechanism that could be responsible for the AuNPs aggregation is the depletion interaction, i.e., the mesoscopic entropic interaction associated with a colloidal system in the presence of polymers that are not adsorbed on the surface of colloidal particles [21–25]. This force arises from the difference of the osmotic pressure between the bulk solution and the zone between the surface of the particles that is polymer-depleted because of increased steric hindrance.

Non-specific protein-DNA interactions are mediated by electrostatic interactions with the charged DNA backbone, hydrogen bonding, van der Waals forces and hydrophobic interactions [62–64]. The whole non-specific interaction can push protein and DNA to interact with a respectable affinity. In our case, displaced BiotinDNA at concentrations estimated in the nM range is able to trigger the aggregation process. Figure 8 shows results from SPR investigation of the BiotinDNA displacement from surface immobilized SA (open circles). Remarkably, BiotinDNA was displaced with a dependence on free biotin concentration similar to that observed for the AuNP-SA-BiotinDNA aggregation, and both experiments reached an almost steady-state condition when 5.12 mM free biotin solutions were used for the competitive displacement.

**Figure 9:**
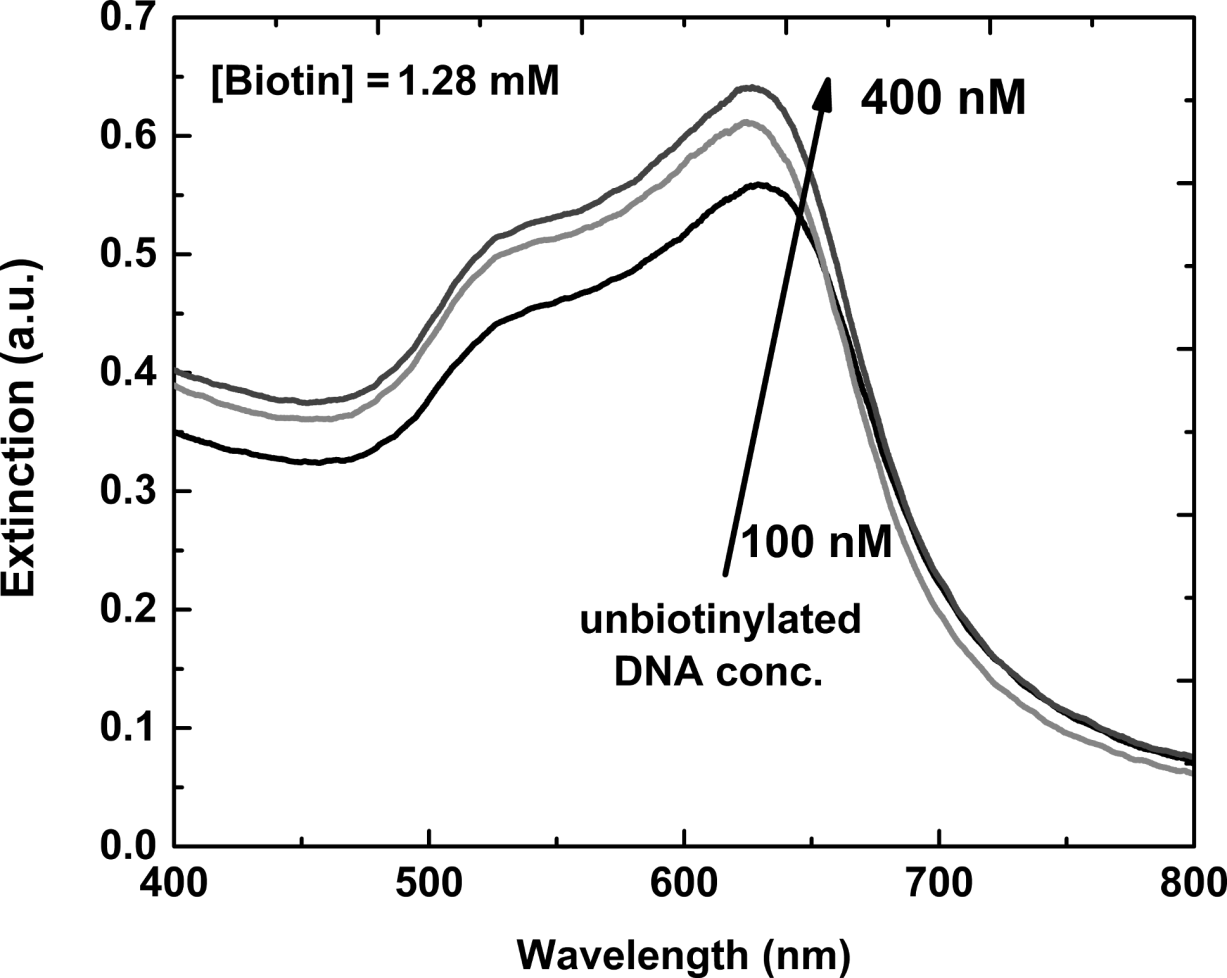
Extinction spectra (400–800 nm) of AuNP-SA in the presence of free biotin (1.28 mM) titrated with unbiotinylated DNA (solutions: 100 nM, 200 nM and 400 nM, respectively).

In order to provide an additional proof of the role played by displaced BiotinDNA molecules on the aggregation of nanoparticles, the behavior of AuNP-SA dispersions in the presence of an increased amount of unbiotinylated DNA was investigated. In particular, AuNPs-SA nanoparticles were incubated with unbiotinylated DNA (final concentrations 100 nM, 200 nM and 400 nM, respectively) in the presence of free biotin (1.28 mM). Figure 9 displays extinction spectra obtained from each solution. An intense plasmon band around 600 nm is obtained, clearly testifying the aggregation of AuNP-SA caused by the added DNA sequences. The latter experiment further proves the role of the oligonucleotide sequence on the aggregation of the nanoparticles. In this case, the unbiotinylated DNA can only non-specifically interact with AuNP-SA due to the absence of the biotin moiety on its structure.

## Conclusion

SA-coated AuNPs were modified with biotinylated oligonucleotide and spontaneously re-dispersed in water. The role of the oligonucleotide in the stabilization of the functionalized nanoparticle dispersion has been investigated by performing a competitive displacement of the biotinylated oligonucleotide through a ligand-exchange process with free biotin. The displacement process detected by adsorption spectroscopy has been confirmed by SPR measurements. Experiments showed the important role played by the displaced oligonucleotide in triggering the fractal aggregation of functionalized nanoparticles, and the difference of the osmotic pressure between the bulk solution and the zone between the surfaces of the particles, that is polymer-depleted because of increased steric hindrance, has been proposed as the driving force for the aggregation process.

Due to the important role played by SA-coated AuNPs in the development of innovative diagnostic approaches we believe that these results will directly impact the ability to produce more reliable and sensitive nanoparticle-based diagnostics assays which could lead to advanced applications in nanomedicine which benefit of the ultrasensitive detection of nucleic acids. In addition, the formation of large aligned one-dimensional aggregates triggered by the displacement of oligonucleotides from functionalized AuNPs has been reported for the first time.

## Experimental

### Materials and reagents

Trisodium citrate dihydrate, tetrachloroauric(III) acid, sodium hydroxide solutions (10 M in water), and biotin were purchased from Sigma-Aldrich (Italy). Biotinylated and unbiotinylated 11-mer oligo deoxyribonucleotide (BiotinDNA, 5’-AGCAGCCTAAG-3’-Biotin, and DNA, 5’-AGCAGCCTAAG-3’, respectively. *T*_m_ = 34.0 °C), successfully employed in previous studies for the detection of non-amplified human genomic DNA [13], were purchased from Thermo Fisher Scientific, Inc. Streptavidin from *Streptomyces avidinii*, provided in lyophilized form in 10 mM PBS, pH 7.4, was purchased from Invitrogen (Italy). Mixed cellulose ester membrane filters were purchased from Whatman (UK). Phosphate buffered saline (PBS) solutions at pH 7.4 (137 mM NaCl, 2.7 mM KCl, phosphate buffered 10 mM) were obtained from Amresco (Italy). Ultra-pure water (Milli-Q Element, Millipore) was used for all the experiments.

### Synthesis of AuNPs

Glassware was cleaned with freshly prepared “piranha” solution; i.e., a mixture of 1:3 ratio of hydrogen peroxide (30%) and concentrated sulfuric acid (98%). Caution: piranha solution reacts violently with most organic materials and should be handled with extreme care. AuNPs were synthesized by citrate reduction of HAuCl_4_·3H_2_O [65]. The trisodium citrate concentration has been shown to be crucial for the preparation and the control of the size of AuNPs [66–67]. Briefly, 20 mL of trisodium citrate (38.8 mM) were quickly added with vigorous stirring to 200 mL of a boiling aqueous solution of HAuCl_4_·3H_2_O (1 mM). The colour of the solution changed from pale yellow to deep red, and a complete reduction was obtained after 10 min. The solution was cooled to the room temperature and filtered through a 0.45 μm mixed cellulose ester membrane filter. To prevent light-induced flocculation of the colloids, all colloidal gold solutions were stored in the dark and refrigerated at 4 °C. Similar conditions assured the nanoparticles stability for several months. The resulting colloidal solution was characterized by λ_max_ = 520 nm.

### AuNPs functionalization

Adsorption of SA on AuNPs was achieved by adding 10 µL of SA solution (1 mg mL^−1^ in 10 mM sodium phosphate buffer pH 7.4, the final concentration of SA in AuNPs solution was 3.6 × 10^−7^ M) to 500 µL of colloidal gold solutions (5 nM, pH 11.4), and the mixture was kept on ice for 1 h. The unreacted excess of SA was removed from the modified gold nanoparticles (AuNP-SA) by centrifugation (Eppendorf 5417R, 30 min, 12500 rpm, 23 °C) followed by decantation of supernatants and redispersion in 90 μL of water. AuNP-SA dispersions were incubated for 30 min with 10 μL of 100 μM BiotinDNA water solution. After centrifugation and removal of the supernatant solution, AuNP-SA-BiotinDNA nanoparticles were dispersed in water. Non-specific adsorption of BiotinDNA on bare AuNPs was achieved by adding 10 µL of 100 μM BiotinDNA to the AuNPs water solutions.

### Characterization of nanoparticles

Optical absorption measurements were performed with Nanodrop^TM^ 1000 and Agilent 8453 spectrophotometers. Spectra were collected in the 200–800 nm range.

Nanoparticles were analysed by using a Zetasizer Nano ZS ZEN3600 (Malvern Instruments, Malvern, UK) instrument equipped with a 4 mW He–Ne laser operating at 633 nm and an avalanche photodiode detector (APD) (quantum efficiency >50% at 633 nm). Measurements were performed at 25 °C by using aqueous AuNP solutions filtered with a 0.45 µm or 0.22 µm pore size membrane. Samples (1 mL) were transferred into a disposable polystyrene cuvette for DLS measurements while a folded capillary cell was used for ζ-potential measurements. The concentration of solutions (0.1–1 nM) was adjusted to accommodate scattering properties of samples and optical requirements of the equipment. It has been found that optimal concentration for DLS worked well also for ζ-potential measurements. The intensity of light backscattered at a detection angle of 173° was used to calculate the mean hydrodynamic diameter (z-average mean) and the overall distribution of particles sizes; i.e., the polydispersity index (PDI).

Electron micrographs of gold nanoparticles were taken with a FEI TECNAI T12 transmission electron microscope (FEI, Hillsboro, Oregon, USA) operating at 120 kV. 2 µL of nanoparticle dispersion were drop cast on carbon-coated 300 mesh copper grids (AGS160-3, Agar Scientific). The drop dried for over 5 h under a fume hood before TEM imaging. The images were bi-leveled in ImageJ (National Institutes of Health NIH, USA) using the default threshold method. To measure the nanoparticles, a built-in routine (ImageJ, Analyze Particles) was used, without separation methods and constraints.

### BiotinDNA displacement from AuNP-SA-BiotinDNA

AuNP-SA-BiotinDNA nanoparticles were dispersed in water (24 nM). The dispersion was divided into 8 equal batches (45 μL each) and incubated individually at room temperature for 3.5 h with 5 μL of biotin solutions prepared in order to obtain a final concentration of biotin in each batch of 1280 μM, 640 μM, 320 μM, 160 μM, 80 μM, 40 μM, 20 μM, and 10 μM, respectively. After the centrifugation, the supernatant was discarded and nanoparticles were dispersed in 50 μL of water.

### SPR measurements

SPR measurements were carried out by using a SensiQ Pioneer equipment from SensiQ Technologies, Inc. (Oklahoma City, USA). Chips for SPR were purchased from ICx Nomadics (Oklahoma City, USA). SA was immobilized at 25 °C on the carboxylated hydrogel matrix of COOH5 sensor chips through the standard amine-coupling method. For this purpose the COOH5 chip surface was activated for 3 min with 0.2 mM EDC (1-ethyl 3-(3-dimethylaminopropyl)carbodiimide hydrochloride) and 0.05 mM NHS (*N*-hydroxysuccinimide), then an SA solution (volume: 100 µL; flow rate: 10 µL min^−1^; concentration: 200 mg L^−1^ in 10 mM acetate buffer, pH 4.5) was flowed through channels 2 and 3 of the SPR fluidic device. 1 M Ethanolamine (pH 8.5) solution was used to deactivate unreacted functional groups.

A 10 µM BiotinDNA water solution was flowed (flow rate of 10 µL min^−1^) through channel 2 and 3 of the SPR fluidic system and adsorbed on SA-functionalized surfaces. The solution was also flowed through channel 1 and adsorbed on a portion of the chip surface where no SA was present. SPR signals (RU) detected from the latter region were used to correct SPR responses from the SA-functionalized surface for refractive index changes, nonspecific binding, and instrument drift. The displacement of BiotinDNA from the surface-immobilized SA was achieved by injecting increasingly concentrated free biotin water solutions (10.24 mM, 5.12 mM, 2.56 mM, 1.28 mM, 0.64 mM, 0.32 mM, 0.16 mM, 0.08 mM, 0.04 mM, 0.02 mM, 0.01 mM).
